# Limited access to family-based addiction prevention services for socio-economically deprived families in Switzerland: a grounded theory study

**DOI:** 10.1186/s12939-020-01305-1

**Published:** 2020-10-28

**Authors:** Andreas Pfister, Nikola Koschmieder, Sabrina Wyss

**Affiliations:** Institute of Social Management, Social Policy and Prevention, School of Social Work, Lucerne University of Applied Sciences and Arts, Werftestrasse 1, Postfach 2945, CH-6002 Lucerne, Switzerland

**Keywords:** Family-based, Substance abuse, Addiction, Prevention, Health promotion, Access to services, Identification of candidacy, Low SES, Deprived families, Hard-to-reach

## Abstract

**Background:**

Families living in poor socio-economic circumstances, already confronted with social and health inequalities, are often not reached by family-based addiction prevention services. Besides quantitative models and health literacy approaches, qualitative research is lacking that could shed light on the exact circumstances and processes that lead to hindered addiction prevention service uptake by these families. Drawing on the concept of candidacy, we therefore reconstructed how socio-economically deprived parents and their (pre) adolescent children in the German-speaking part of Switzerland (non-)identified their candidacy for family-based addiction prevention services.

**Methods:**

Following grounded theory, we collected and analysed data in an iterative-cyclical manner using theoretical sampling and theoretical coding techniques. Sixteen families with children aged 10–14 years were interviewed in depth (parent/s and child separately). All but one family lived below the at-risk-of-poverty threshold.

**Results:**

Socio-economically deprived families’ modes of recognizing and handling problems in everyday life were found to be core phenomena that structure the process towards (non) identification of candidacy for family-based addiction prevention services. Four modes anchored within socio-demographic resources were found: Families with mode A perceived their current life situation as existentially threatening and focused daily coping on the main pressing problem. Others (mode B) perceived prevalent multiple problems as normal (now); problems were normalized, often not recognized as such. In mode C families, problems were pragmatically recognized at a low threshold and pragmatically dealt with, mostly within the family. In mode D families, problems were constantly produced and dealt with early by the worried and anxious parents monitoring their child. From modes D to A, vulnerability increased concerning non-identification of candidacy for family-based addiction prevention services. Further, thematic relevance of addiction prevention, past experience with offers, integration in systems of assistance, strategies to protect the family, and families’ search for information influenced whether identification of candidacy took place.

**Conclusions:**

Socio-economically deprived families differ in modes of problem construction and handling in everyday life; this differently opens up or closes routes to family-based addiction prevention. Addiction prevention practice should build on a bundle of diverse strategies for outreach to these families, stressing especially interventions on the structural and environmental level.

**Supplementary Information:**

The online version contains supplementary material available at 10.1186/s12939-020-01305-1.

## Abstract (de)

**Ausgangslage:**

Sozioöonomisch benachteiligte Familien, bereits mit sozialer und gesundheitlicher Ungleichheit konfrontiert, werden von Massnahmen familienbezogener Suchtprävention oftmals nicht erreicht. Neben quantitativen Modellen und Forschung zu Gesundheitskompetenz (*Health Literacy*) fehlt es an qualitativer Forschung, die Aufschluss über die genauen Umstände und Prozesse geben könnte, die zur erschwerten Inanspruchnahme von Angeboten durch diese Familien führen. Ausgehend vom “Concept of Candidacy” rekonstruierten wir deshalb, wie sozioökonomisch benachteiligte Eltern und ihre (prä-)adoleszenten Kinder in der Deutschschweiz ihre Kandidatur für familienbezogene Suchtprävention (nicht) identifizieren.

**Methoden:**

In Anlehnung an die Grounded Theory erhoben und analysierten wir Daten iterativ-zyklisch mittels theoretischem Sampling und theoretischem Kodieren. Sechzehn Familien mit Kindern im Alter von 10–14 Jahren wurden mittels problemzentriertem Interview befragt (Elternteil/e und Kind getrennt). Alle bis auf eine Familie lebten unterhalb der Armutsgefährdungsschwelle.

**Ergebnisse:**

Die Art und Weise, wie sozioökonomisch benachteiligte Familien Probleme im Alltag erkennen und bearbeiten, erwies sich als Kernphänomen, das den Prozess zur (Nicht-)Identifizierung als Kandidat/in für familienbezogene Suchtprävention strukturiert. Vier Modi, verankert in unterschiedlichen soziodemographischen Ressourcen, wurden gefunden. Familien mit Modus A empfanden ihre aktuelle Lebenssituation als existenzbedrohend und fokussierten daher die Alltagsbewältigung auf das drängende Hauptproblem. Andere Familien (Modus B) nahmen den mit multiplen Problemen belasteten Alltag (mittlerweile) als normal wahr. Probleme wurden normalisiert, oft nicht als solche erkannt. In Modus-C-Familien wurden Probleme niedrigschwellig pragmatisch erkannt und pragmatisch bearbeitet, meist innerhalb der Familie. In Modus-D-Familien wurden Probleme durch den besorgten und ängstlichen Zugriff der Eltern stetig hervorgebracht und früh bearbeitet. Von Modus D hin zu A erhöhte sich das Risiko, dass die Familien sich nicht als Kandidaten für familienbezogene Suchtprävention identifizierten. Darüber hinaus beeinflussten die thematische Relevanz von Suchtprävention, frühere Erfahrungen mit Angeboten, die Integration in Hilfesysteme, Strategien um die Familie zu schützen und die Suche der Familien nach Informationen oder Unterstützung, ob eine Identifizierung mit Angeboten zu Stande kam.

**Schlussfolgerungen:**

Sozioökonomisch benachteiligte Familien verfügen über unterschiedliche Modi, Probleme im Alltag zu erkennen und zu bearbeiten; dadurch öffnen beziehungsweise schliessen sich unterschiedlich die Wege hin zu familienbezogener Suchtprävention. Health in All Policies und die suchtpräventive Praxis sollten deshalb unterschiedliche Strategien – insbesondere strukturelle und umwelbezogene Zugänge – anwenden, um diese Familien zu erreichen.

## Background

Socio-economically deprived population groups face barriers to accessing health (promotion) and prevention services [1–5]. Socio-economically deprived families’ also face barriers to accessing family-based addition prevention services (FAPS) [6, 7]. Moreover, international research demonstrates that children living in socio-economically deprived families are more at risk for behavioural difficulties and substance use/abuse in adolescence than children in more affluent families [8–11]. Therefore, especially socio-economically deprived families should be effectively involved in FAPS. Health promotion and prevention activities *not* reaching these families would further accentuate already existing social and health inequalities, which is not acceptable from an equity in health perspective. However, there is a lack of international as well as Swiss studies on the exact circumstances of hindered access to FAPS for socio-economically deprived families. Therefore, the aim of our study was to close this knowledge gap, to qualitatively reconstruct limited access to FAPS for socio-economically deprived families with (pre) adolescent children, in order to identify strategies to improve equity in access to FAPS for this population group. We posed the following research questions:

- How do socio-economically deprived parents and their (pre-)adolescent children become candidates (identify their candidacy) for FAPS in the German-speaking part of Switzerland?
- Based on what constellations and (life) circumstances do parents and children conclude (or not conclude) that information about addiction prevention, offers of help, and/or contacts to specialists (e.g. social workers) are relevant for them and worth considering?

### Introducing FAPS and the context of (German-speaking) Switzerland

Adolescence is a window of opportunity for shaping health behaviour [12]. Risk and protective factors in families that impact adolescent’s substance use behaviour are well known [6, 13, 14], such as effects of parenting skills and parental monitoring on adolescents’ substance use [11, 15–18]. Therefore, preventive efforts such as FAPS are directed towards parents and families. Even though there is evidence that children in socio-economically deprived families are more at risk for future substance abuse (see above) and that – as found in a sample of Swiss adolescents – a high level of parental monitoring decreasing adolescents’ substance use is associated with high socioeconomic status [11], all families are addressed by FAPS. All parents are important resources for the health of their children. Universal prevention activities target all families (e.g. all parents of students in a school class) irrespectively of their potential risk factors, socio-economic status (SES), or level of risky (substance) use patterns. Selective prevention targets families with existing risk factors (e.g. parents with low SES and/or with addictions) that increase the probability of future substance use/abuse in children living in these families. The overall goals of FAPS on the universal and selective level are to prevent or delay the initiation of (substance) use, “… or to reduce the frequency or volume of use among children of participating parents” [19]. Indicated interventions intend to reduce already prevalent risky (use) patterns among children of participating parents.

The extent and form of FAPS can vary [7, 14, 19]. The services can be directed towards parents exclusively, children and parents (e.g. school-based), or – besides parents and children – diverse parties, such as a wider population in a city quarter, sport clubs, etc. [7]. “[M]ost existing programs however target parents and do not include the adolescents” [14]. Many programmes do not focus exclusively on substance or excessive media use but also address and promote wider familial resources and competencies, such as parenting skills, parental monitoring, and life skills [14, 19], because FAPS are more effective when these broader familial resources are addressed [14].

A 2012 national report on FAPS in Switzerland concluded that FAPS on a universal level are widespread, but FAPS on selective and indicated levels are much rarer [7]. This is also the case for the German-speaking part of Switzerland. In most cases, parents are addressed on a universal level in school or leisure settings (e.g. parent night on addiction prevention). All families have the same rights (e.g. irrespectively of residence status) to access FAPS. Participation is voluntary and no monetary costs arise for participants. But parents (and children) have to make their way to schools and event locations. Transportation costs, time spent until arriving at the event location, and overall time spent during the event, arise (direct and indirect costs). For universal FAPS, all families are eligible, whereas selective and indicated FAPS target families with risk factors (selective prevention) or already prevalent risky consumption patterns (indicated prevention) in adolescents (see above). In indicated and selective preventive activities, based on risk assessments, social workers, teachers, school psychologists try to include families at risk (voluntary participation!). Independently, these families can also directly contact indicated and selective offers and the above-mentioned professionals involved. FAPS take place in a national context (Switzerland) in which in 2018, according to the Federal Statistical Office, 7.9% of the permanent resident population in Switzerland were affected by income poverty [20]; 13.9%, or almost every seventh person, was at risk of poverty in the same year [21]. Although the poverty rate, persons affected by income poverty, fell from 9.3 to 5.9% from 2007 to 2013, the rate has been rising again since then ([22], p. 56). Families, especially single-parent households and couples with two or more children, are particularly affected [21]. Thus, FAPS need to reach this relevant and important target group of socio-economically deprived families.

### Existing barriers and identification of candidacy for services

Research on parental engagement in preventive parenting programmes is limited [6, 23–25]. Specifically for FAPS, Laging [6] compiled factors that affect willingness to participate in these programmes, differentiating between intra-family and programme organizational factors. Intra-family factors are cognitions (e.g. privacy concerns [26]), extent of family conflicts, parenting and communication styles, level of order and organization, and the influence of individual family members that are not willing to participate. Some studies found that families most likely to benefit from such programmes are less likely to participate [27–29]. On the programme organizational level, logistical barriers and pragmatic aspects (childcare, costs, transport, time, place, programme duration), gratuities for participants, involvement of schools and communities (active promotion), and programme coordination and support were identified as relevant for participation ([6], p. 10). But, due to the state of research, it was not possible for Laging [6] to set the intra-family and programme organizational factors in relation to families with socio-economic deprivation. We still do not know how, exactly, especially also from the perspective of the families concerned, limited access to FAPS comes about in these families. Existing general models and theories explaining health behaviour and health services use [30–35] and health literacy research [36–38] are very useful in understanding access to health services and frequently pinpoint SES. Levesque et al. [39], for example, conceptualise access in a patient-centred view at the interface of health systems and populations and integrate supply and demand-side factors. But, these models are mostly quantitative and – with the exception of Levesque et al. [39] – rather static. Besides identifying relevant factors (also SES), they cannot shed light on the exact circumstances and processes that result in families with socio-economic disadvantage being poorly reached by FAPS. Therefore, and also as qualitative research methods are generally underrepresented in health services use research [2, 40], we found that the concept of *candidacy* can aid a better understanding of (limited) access to and utilization of FAPS by socio-economically deprived groups.

The concept of candidacy emerged from a “critical interpretive synthesis” of the scientific literature and empirical studies on access to healthcare by vulnerable groups in the UK [1, 5]. It has been considered useful for understanding the journeys of vulnerable groups not only through different kinds of health services [41–51] but also through public services in general [52]. The concept of candidacy draws on an interactionist and process-oriented perspective and sees access to services as influenced by simultaneously ongoing processes determined by users and health services. According to Dixon-Woods et al.:

“Candidacy describes how people's eligibility for healthcare is determined between themselves and health services. ( … ) Health services are continually constituting and seeking to define the appropriate objects of medical attention and intervention, while at the same time people are engaged in constituting and defining what they understand to be the appropriate objects of medical attention and intervention. Access represents a dynamic interplay between these simultaneous, iterative and mutually reinforcing processes. By attending to how vulnerabilities arise in relation to candidacy, the phenomenon of access can be better understood, and more appropriate recommendations made for policy, practice and future research.” ([1], p. 1)

The candidacy journey through health services consists of seven stages: identification of candidacy; navigation of services; permeability of services; appearing at services and asserting candidacy; adjudication by professionals; offers of, resistance to, services; operating conditions and local production of candidacy ([52], p. 809). Identification of candidacy, the “process by which individuals come to view themselves as legitimate candidates for particular services” ([52], p. 809), is the starting point of access and plays a key role in facilitating or hindering access of (vulnerable) groups to services. Within socio-economically deprived communities, Dixon-Woods et al. found that managing “health as a series of minor and major crises”, potentially due to normalization of health problems, affects identification of candidacy for health services negatively, especially for prevention services ([5], p. 103). Due to the importance of (potential) users’ identification of candidacy for gaining access to prevention services as well as the predominant focus in health services access research on facilitators and barriers in health systems and services level (see also [39]), we decided to focus our research on users for a better understanding of how socio-economically deprived families in the German-speaking part of Switzerland identify their candidacy for FAPS.

## Methods

Grounded theory following Strauss and Corbin [53] provides a well-proven set of qualitative-interpretative procedures for developing theory from data, especially in new and emerging research areas. We therefore concluded that grounded theory [53] and the concept of candidacy as a “sensitizing concept” [54] would fit our research purposes best. According to Blumer, the benefit of using a sensitizing concept in empirical social research is not to prescribe what to see to researchers, but to “… merely suggest directions along which to look” ([54], p. 7). The use of sensitizing concepts is common practice in grounded theory studies [55–57]. A completed COREQ checklist (consolidated criteria for reporting qualitative research) can be found in Additional file 1.

### Data collection

From May 2017 to January 2020 we conducted and analysed 32 interviews with socio-economically deprived families in the German-speaking part of Switzerland. Following a theoretical sampling strategy [53], we alternated phases of data collection and analysis. This iterative procedure allowed us to be driven by concepts identified in and raised by empirical data and to move towards a saturated grounded theory.

#### Inclusion criteria and sampling procedures

To include all forms of families (e.g. single parents, two-parent families, same-sex couples with children, etc.), we defined ‘family’ as a social community consisting of at least one child and at least one adult, in which the adult(s) has/have a caring role towards the child. Families were included in the study that:

- consisted of at least one child aged 10–14 years
- lived in the German-speaking part of Switzerland
- had less than 60% of the national median equivalized income (= at-risk-of-poverty threshold [58]) at their disposal

The ages 10 through 14 are formative years in developing health behaviours [12], particularly concerning first interest in and potential use of psychoactive substances [59–62]. For that reason, families with children aged 10–14 are often targeted by FAPS and were thus of key interest for our study.

The family’s socio-economic status (SES) was determined by parents’ income, education, and occupational status [63]. As recent research draws attention to the diversity of families with socio-economic deprivation [63, 64], using the at-risk-of-poverty threshold [58], we only defined family income as hard inclusion criteria concerning SES. In line with theoretical sampling, we included also families with inconsistencies in SES (for example, higher educational backgrounds) in order to get a fuller picture of socio-economically deprived families. Taking into account different walks of life and different backgrounds, for example in terms of family size and constellation, parents’ and child’s age, ethnicity, and gender, and based on concepts and questions that the analysis of previous data offered, the overall research team decided continuously what type of family to include next in the study. Drawing on grounded theory methodology, we hereby applied strategies in maximizing and minimizing contrasts [53] (e.g. including a family that lived above the at-risk-of-poverty threshold).

#### Recruitment of participants

How can a population group that is already hard-to-reach for prevention and health promotion services be included successfully in a study? Drawing on methodological literature [65], we resolved that challenge by carefully thinking through effective recruitment strategies and potential biases that could arise from applying strategies (e.g. by exclusively recruiting within the social services sector).

In several German-speaking cantons of Switzerland, we (two women and one man) directly approached adults on the street that could potentially be parents of a child aged 10 to 14 or that were accompanied by a child of that age. During this first contact we gave the information that we would like to learn more about the everyday life of families that have to get by with little money. One or two researchers were present on site during the recruitment. We did not profile people, did not selectively approach people whom we thought were poor, but approached all adults on the street that could potentially be parents. Guided by national and regional statistics, we were present in less affluent city quarters, in front of grocery stores that are limited to people with low socio-economic status, or in front of second-hand shops. We also asked persons to spread the information among their acquaintances. Every time we visited sites and locations, we placed and distributed flyers and sensitized persons at the site for our study. Further, we recruited online, placed study information and digital flyers in Internet forums for parents, and maintained a Facebook profile. Occasionally, we collaborated with mediators from the social sector. During the research process and onward theoretical sampling, we needed to also include families that made their way to FAPS in order to be able to qualitatively reconstruct processes of successful candidacy for FAPS. We therefore presented our study and distributed flyers at a parent night on addiction prevention.

When both parent/s and their child aged 10 to 14 consented to participate in the study, primary information was gathered: number of persons in the household, number of children and their age, parents’ educational attainment, occupational status (incl. Percentage of full-time work), and monthly net household income in the household in which the child mainly lived. When families met hard inclusion criteria, and depending on the status of the theoretical sampling, the respective family was either included in the study right away or was put on a waiting list.

#### Problem-centred interviewing

Interviews were conducted following Witzel’s problem-centred interview, which is a loosely structured in-depth interview [66, 67]. Parent/s and the child were interviewed separately because of the sensitive issue and to avoid biased narrations (e.g. to avoid child stating no interest in substance use in front of parents, despite first user experiences). The interviews with the parents lasted on average 93 min (range 34 to 157 min), the interviews with the children 57 min (range 39 to 83 min). Prior to the interview, researchers informed parents and children about the study (goals, data handling, etc.) and both parent/s and the participating child gave oral consent to participate. Parents moreover provided written consent (also for their minor child). The authors of this study conducted parent interviews either with one parent or whenever possible with both parents. Interviewed families were free to choose the place for the interview (e.g. at their home, at a cafeteria). The authors are trained and experienced interviewers; they hold either a Master of Arts in sociology or a PhD in educational sciences/social pedagogy. The interviews were conducted in Swiss German, German, or French. Two native-speaking university students in Social Work and Teaching conducted interviews in Tamil and Kurdish with non-German-speaking parents and translated the interviews into German. The students were trained and supported by two of the authors.

The interview guides can be found in Additional file 2 (parent interview guide) and Additional file 3 (child interview guide). Following Witzel’s problem-centred interview technique, our opening question aimed at encouraging narratives ([67], p. 68): “I am interested in how you live. As a family, what do you do all day? What are you engaged in? Please tell me about it.” We then asked the parents about their everyday life and about the child’s development (incl. Substance use experiences) and health. We also asked parents about their knowledge and experience regarding FAPS and other support offers. When interviewing the children, we started with warm-up questions and asked about their age, class level, and leisure activities. Afterwards, the opening question, posed in a child-appropriate manner, invited the young respondents to talk about their day and what they were generally engaged in. Then, always following child’s narrative, the overall same topics were covered as in the parent interview. At the end of the child and the parent interview, we used a semi-standardized short interview questionnaire to record socio-demographic characteristics. After interview completion, parents and the child received a small expense allowance (in form of a voucher for daily products). We then noted down the circumstances of the interview (e.g. setting of the interview, interactions between interviewer and interviewee) in a postscript.

### Participant characteristics

The final sample consisted of 16 families (20 interviewed parents, 16 interviewed children) that resided in rural and urban areas (incl. Urban agglomerations) in the German-speaking part of Switzerland in the cantons of Basel-Stadt, Bern, Lucerne, Nidwalden, Obwalden, St. Gallen, and Zurich. All but one family lived below the at-risk-of-poverty threshold [58] (see all participant characteristics in Table 1).

**Table 1:** Study sample (*n* = 36 interviewed persons)

			Total
**Parents (*n*** **= 20)**	Gender	Female	14
		Male	6
	Age in years	30–35	3
		36–40	7
		41–45	6
		46–50	3
		51–55	–
		56–60	–
		61–65	1
	Educational attainment^a^	No compulsory education	3
		Compulsory education	1
		Upper-secondary level (vocational education and training)	13
		Tertiary level (professional education)^b^	3
	Country of birth	Switzerland	9
		Other^c^	11
**Children (*n*** **= 16)**	Gender	Female	10
		Male	6
	Age in years	10	4
		11	2
		12	6
		13	1
		14	3
**Household (*n*** **= 16)**	Type of household	Couple (married or living together) with child (ren)	8
		Single parent with child (ren)	8
	Number of children in household	1	5
		2	4
		3	4
		4–6	3
	Financial situation	Above the at-risk-of-poverty threshold	1
		Income is about 60% of the median equivalent	5
		Income is 50% or less of the median equivalent income	10
	Main source of income	Employment	11
		Social insurance (old age or disability pensions)	2
		Social welfare	3

^a^ In the Swiss Vocational and Professional Education and Training system ([70], p. 6).

^b^ Some of the tertiary education qualifications were acquired abroad and were not accepted in Switzerland.

^c^ Five of them now have Swiss citizenship.

### Data analysis

The interviews were audio recorded, fully transcribed, and coded using MAXQDA software. We anonymized the transcripts and safely stored the data on a Swiss university server. The coding strategy followed the guidelines of grounded theory [53]. As suggested by the approach, the analysis oscillated between inductive and deductive proceeding, continuously asking questions and making comparisons. We started with open coding and gradually built, tested, reviewed, and enriched concepts to answer our research questions (axial and selective coding). After coding the parents’ and the child’s interview, we set them in relation to each other in writing a case characterization. This helped us to understand candidacy processes in the overall family unit. Every interview was coded in minimum by two of the present authors. Case characterizations were always discussed and reviewed by the overall research team (three persons). The concept of candidacy served as a sensitizing concept [53, 54] throughout this process. At final stage of the analysis, in a daily workshop with two external social work professors and one specialized addiction prevention practitioner, we discussed and validated the preliminary findings. The aim was to discover potential blind spots in the analysis and to ensure scientifically sound results that can instruct policy and practice.

## Results

**Fig. 1:**
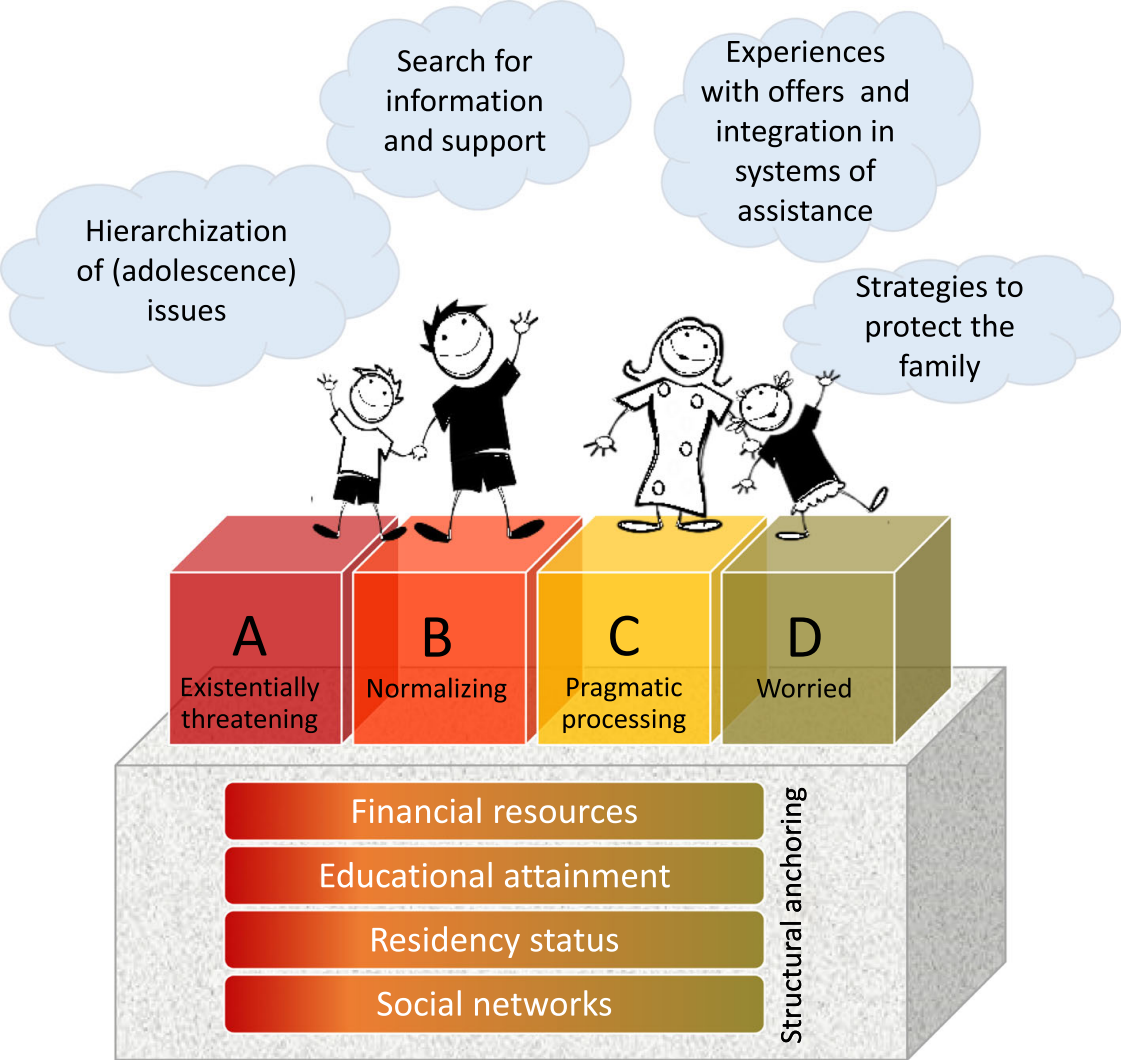
Grounded Theory on socio-economically deprived families' limited access to FAPS

Our grounded theory (see Fig. 1) on socio-economically deprived families’ limited access to FAPS is as follows: Depending on families’ modes of recognizing and handling problems in everyday life, routes towards identification of candidacy with services/offers tend to be furthered (in green) or hindered (in red). The modes are structurally anchored in different resources, from weak (in red) to stronger resources (in green), and the modes’ effects on identification of candidacy for FAPS are weakened or strengthened by other influencing factors (see clouds in Fig. 1).

### Mode of recognizing and handling problems in everyday life

As the way problems were recognized and handled determined not only what was seen as a problem but also when and what kind of action was required, families’ modes of recognizing and handling problems in everyday life were found to be core phenomena that structure the process towards (non-)identification of candidacy for FAPS. We identified four different modes in the data:

- Mode A – *existentially threatening*: The family’s current life situation was perceived as existentially threatening. The focus of coping was on the problem perceived as existentially threatening.
- Mode B – *normalizing*: The burden of multiple problems in the family’s everyday life was perceived and experienced as normal (now). Problems were normalized, often not recognized as such.
- Mode C – *pragmatic processing*: The family’s everyday life continued on, despite financial precariousness. Problems were pragmatically recognized at a low threshold and dealt with pragmatically (mostly within the family).
- Mode D – *worried*: Problems were constantly produced in everyday life by worried and anxious parents monitoring their child. Problems were dealt with at an early stage (low problem threshold).

The modes are not exclusive categories. All families analysed had a main mode, but they sometimes also applied strategies that were prevalent in (an) other mode(s).

#### Mode A: existentially threatening

Some families saw their existence threatened by one main problem: *“I said there’d be money today. I checked. It hasn’t come yet. I only have 2 francs... I bought potato chips for the children”* (NH, mother, age 36). A high level of financial precariousness, unsecured residence status (e.g. provisionally admitted foreigners – Permit F), or a single father’s incompatibility of poorly paid shift work (working poor status) with caring for his child were such existentially threatening problems.

Parents’ perception and identification of a main existential burden in their daily life created a specific pressure to cope with that issue first, because it was seen as the origin of all misery: *“… you know, I think if we get a residence permit, all my problems are over”* (GAA, father, age 50). Therefore, a specific and thematic focus on action and coping was set in these families. All other issues were regarded as secondary or as a consequence of the existentially threatening problem. As a result, few resources were freed up for dealing with other problems and issues on a day-to-day basis. Topics in the area of addiction prevention such as substance use, media use, or parenting skills were subordinated to the processing of and coping with the situation perceived as existentially threatening. Hence, health issues and problems were only perceived (if at all) when they were very acute (high problem threshold), which turned out to be a barrier to identification of candidacy for (addiction) prevention services. Moreover, parents with this mode of problem construction and problem handling, in contrast to modes C (pragmatic processing) and D (worried), seemed to have low agency. Probably caused by multiple deprivations and problem load, they preferred support for their children and not for themselves (e.g. in order to better support their child or resolve the problem on their own). Hence, they identified more easily with offers for children than with offers for parents, which led to non-identification of their candidacy for FAPS.

#### Mode B: normalizing

Some families, even though they were confronted with multiple problems in everyday life (e.g. financial deprivation, parent’s mental illness, child’s psychosocial problems), perceived this situation as normal rather than exceptional. For example, the aggressive behaviour of a 10-year-old son who had beaten his mother was framed by his mother as a kind of normal, male behaviour. Another mother felt there was no need for support (professionally or by herself) for her daughter after her ex-partner’s (the girls’ father’s) second suicide attempt. The situation was normalized. The daughter just had *“… to go through it, he is her father, you know”* (SS, mother, age 44), the mother told us.

As demonstrated, the threshold to perceive something as posing a need for action was high. Circumstances that would be constructed as problematic in other families were not perceived that way and were therefore not relevant enough for the family to take action to change the situation. Many issues were accepted, relativized, or normalized, if they were not highly urgent, visible, and/or new to the family. This was also the case concerning health issues. A health problem usually became an issue for the family only when it was acute and visible, for example when there were clear, acute complaints or signs on the body: *“… a little bad what I saw, she [the daughter/AP] has this thing with the... wrist cutting - I went with her to a counsellor”* (SP, mother, age 44). (Health) issues that did not become visible and a problem, that did not exceed a certain problem threshold, received little attention. Therefore, mode B was generally a barrier towards identification of candidacy for addiction prevention services. As in mode A (existentially threatening), also here we reconstructed that the parents had low agency. If support was actually sought and received, it was mostly for their children and not for the parents in order to better support the children. This led to non-identification of candidacy for FAPS. However, by means of professional services provided to children, some children got in contact with health-promoting and life skill-oriented offers that were not family-related (non-participation of parent/s).

#### Mode C: pragmatic processing

In the families with mode C, everyday life took a more or less orderly course despite resource constraints. Issues and problems emerged from the surface of everyday family life, were noticed, and received attention. Problems and relevant topics were constantly discovered by the families, taken up pragmatically, then processed and worked through, one after the other. We therefore called this mode ‘pragmatic processing’. Conversations between parents and children were found to be a favoured strategy for tackling issues, as the following comments by a mother to her daughter illustrates: *“… and when that [menstruation/AP] comes, you don’t have to be afraid, you can come to me, then we’ll talk about that”* (AK, mother, age 35). Topics of prevention, also within the area of addiction prevention (media literacy, parenting skills), came into focus if parents observed a discrepancy between their conceptions and their children’s behaviour, or if children brought up an issue. Thus, due to the recognizing of problems in mode C, paths to identification of candidacy for FAPS generally opened up. However, due to the problem handling strategies in mode C, identification of candidacy with addiction prevention services in these families often did not come about. These families drew a clear line between inside and outside the family. They relied mostly on themselves (‘We’ll find a solution by ourselves’) and on their private network when resolving problems. Equipped with high agency and overall high problem-solving capabilities, these families had a high problem threshold before accepting (professional) help or service offers from outside the family, also concerning FAPS. But the data material showed that also pragmatic-processing families could identify their candidacy for addiction prevention services. Parents who saw it as a matter of course to participate in parents’ events and who were firmly integrated in a (help) system, e.g. were part of a parents’ council, attended educational and information events for parents regardless of the topic (see ‘ Experiences with offers and integration in systems of assistance’ below).

#### Mode D: worried

In families with mode D, relevant topics and (possible) problems were regularly produced in everyday life. This was due to the parents’ worried and sometimes anxious approach to reality and daily living. In one of the interviews this became evident in that the mother mentioned her worries over 17 times (e.g. worries about her son’s safety, his emotional sensibility, or his future). The duality of worrying and caring for children is apparent with these parents; as one mother puts it: *“Well, I am rather the worried mother, I try to prevent all kinds of things”* (KG, mother, age 34). These parents monitor their child intensively in order to promote the child’s psychosocial development and to provide the best possible future for their child.

The way that problems were actively constructed and produced within the family was the reason why even if (possible) problems were not yet present, these parents – in our data, mothers – by definition were very sensitive in everyday life and had an impulse to act on before something became a problem. These mothers therefore quite easily identified the family’s candidacy for addiction prevention services and other health-promoting offers.

In contrast to many families with mode C (pragmatic processing), these families were less oriented towards the inner family circle in their approach to preventive issues and dealing with (potential) problems. They actively and broadly put out feelers for supporting and encouraging information and offers in their social environment. For them, it was part of the normal case, part of their educational self-image, to constantly make use of offers, no matter what topic, and to independently process the knowledge that they acquired: *“Because (3) no matter what, you learn something. Even if it is bad. I see things this way (2). [...] I register for all seminars, everything that is offered by the parents’ council and that comes from the school and I really go everywhere. There is a private school [...], they often have free offers for people and I go there too, just to listen. And sometimes there are topics that don’t concern me at all [ …*]. *But still better to go and listen than (1) not participating in anything”* (IR, mother, age 44).

These parents, in contrast to parents with modes A (existentially threatening) and B (normalizing), did not exclusively search for offers for their children or put their effort into resolving problems on their own (mode C, pragmatic processing). Drawing on strong agency as parents, parents with mode D (worried) sought support, information, and help in addiction preventive and health-promoting offers in order to better support and educate their child. FAPS, which aim to strengthen and develop the skills of parents, therefore fitted the needs and the habitus of these parents.

#### Structural anchoring of the modes

Socio-demographic and structural references became apparent in the data material, which served as the basis for the shaping of the modes (see Fig. 1). If one had to draw a line between the four modes A to D that separates families with a high level of and sometimes multiple resource deprivation from families with slightly better resources, the line would run between two groups, A/B (existentially threatening/normalizing) and C/D (pragmatic processing/worried). In A (existentially threatening) and B (normalizing) there were mainly families with very low educational attainment (even no school-leaving certificate, illiteracy) and low occupational status. Some of them were only provisionally admitted foreigners in Switzerland (Permit F). In A (existentially threatening), the financial resources were very weak. All families were social welfare recipients. In B (normalizing) there were families receiving social welfare as well as unemployed parents (including those receiving a part of a pension from the Swiss invalidity insurance). Some families with mode B were characterized by multiple deprivations and the burden of having many problems and challenges at the same time (financial, social, and health related). In contrast, families with modes C (pragmatic processing) and D (worried) were generally slightly better off financially. Almost all of them had educational attainment ranging from basic vocational education and training (e.g. apprenticeship) to professional education/tertiary degrees (also from abroad, which were not formally accepted in Switzerland!). It is further noticeable that many of them had lived in Switzerland for a longer period of time (compared to families with mode A, existentially threatening, with a shorter period of residence) and/or had Swiss citizenship. Moreover, social networks tended to be better developed in families with modes C/D (pragmatic processing/worried) than with modes A/B (existentially threatening/normalizing).

### Hierarchization of (adolescence) issues: addiction prevention at the bottom

A hierarchization of problems as seen in the described modes, impacting identification of candidacy for FAPS, takes place also concerning issues that parents think are relevant and important for the development of their (adolescent) child. Addiction prevention issues were in general deemed less important than other problem burdens and educational issues related to adolescence. The parents were mostly concerned about their child’s education and school performance, the child’s physical changes and sexual development, new financial demands from the child (new clothes, shoes, etc.), and the child’s increased autonomy, raising parental questions about safety.

All of the parents assessed their child’s interest in psychoactive substances as either non-existent or not problematic, even though children sometimes displayed interest or even indicated (first) substance use in the interviews. The parental (mis) perception that their child was not yet concerned with substance use was therefore an important prerequisite for non-identification of their candidacy for FAPS. The parents based their perception of the child’s non-interest in substance use on the following aspects:

- Child’s stage of development, believing that the child was too young or too far removed from the subject: *“… but I have a feeling she’s still a little far from that [substance use topics/AP]. ((yes)) Well, she’s not that interested yet. But I know this is something that’s sure to come”* (VS, mother, age 35)
- Child’s explicitly expressed disinterest in substance use in conversations with parents
- Child having no friends who used substances (assessment of the peer environment)
- Gender-biased risk assessment, thinking that daughters were less at risk for using psychoactive substances than sons

Parents were more critical and sensitized to children’s media use (e.g. by identifying excessive smartphone use) than to their substance use. Content-wise identification of candidacy for FASP tackling media use was easier than for FASP tackling substance use/abuse, parenting, or life skills.

### Experiences with offers and integration in systems of assistance

The interviews revealed that negative experiences within a system of assistance potentially hindered further contacts with this specific system, whereas positive experiences facilitated contact; the positively evaluated system of assistance was then often the first reference point when families were confronted with further problems or unresolved questions.

A strong anchoring within governmental institutions that provide social welfare or advice concerning migration (mostly families with mode A, existentially threatening, and B, normalizing) turned out to be more of a barrier to than a resource for identification of candidacy for FAPS. Because these institutions and professionals dealt exclusively with specific, acute, and urgent problems (e.g. providing social welfare, residence permit, etc.), there was no triage of these families to health promotion-related and addiction prevention-related offers by these professionals or institutions.

A firm connection to aid organizations and educational institutions, such as school, parents’ council, community centres, charitable organizations, and so on turned out to facilitate the identification of candidacy for FAPS, as long as the providers conducted addiction-preventive courses. When once integrated in a system and firmly connected to it, families took part in offers from these institutions as a matter of course, regardless of the topic: *“I register for all seminars, everything that is offered by the parents’ council and that comes from the school and I really go everywhere”* (IL, mother, age 44).

### Strategies to protect the family

Some families used strategies to protect, in some cases even showcase, their role as parents or their family’s image to the outside world. Proactive protection strategies furthered the identification of their candidacy for FAPS, whereas defensive strategies hindered their identification of candidacy for these services.

Several parents with modes C (pragmatic processing) and D (worried) applied proactive protection strategies. These parents expected that participation in parent events would have a positive effect on their image as a family or as parents. Therefore, they identified with an offer regardless of the relevance of the topic because they expected to protect or even booster their image by participating. When S. M., a single mother of two children, was asked why she took part in the parent night on psychoactive substances, she answered: *“I just thought that if I didn’t go, it would look like I were a bad parent”* (SM, mother, age 43).

Parents using defensive strategies were aiming to protect their family’s or the parent’s image by staying away from FAPS. First, some parents with modes A (existentially threatening) and B (normalizing) feared that an interest in certain topics could be seen as an indication of problems in the family. Second, several families – with modes A (existentially threatening), B (normalizing), and C (pragmatic processing) – avoided certain places or persons, regardless of the topic of the event. Based on their often negative experiences, these families assumed that their image was questioned or even threatened in these places or by certain persons: *“I have very little contact with the parents [...] And I have no relation to the state school. So this is really difficult for me. Even after six years. I can’t find any common ground with them. [ …*] *Even with the teachers. I have a completely different opinion than them”* (VS, mother, age 35).

### Parents’ search for information or support

Search for information or support mostly took place in families’ already established help systems (see above). In active search movements, the search process was activated and guided by a current concern in the family that was demanding attention (often financial issues). Passive search movements were characterized by the fact that they were not initiated by a concern demanding attention and a deliberate search decision. Instead, it was because of parents’ general interest (not limited to financial assistance) that initial information on offers (e.g. flyers, advertisements, etc.) was perceived by the families and search movements were initiated: *“I’m the kind of person who collects information and hangs it on the wall. [giggles] Yes, and just on occasions I look at it and look at the date and sometimes I think, hey, that evening I have time to do something. [ …] Well, I took a few courses like that”* (KG, mother, age 34).

Whereas general interest and the willingness and time to receive and consider information from providers formed the bases for passive search movements and could lead to identification of candidacy for FAPS, active search movements were mostly a barrier to identification of candidacy for addiction prevention due to the focus on concerns demanding attention (e.g. financial assistance). Here again, families with mode A (existentially threatening) were the most vulnerable regarding non-identification of candidacy.

## Discussion

Our results contribute to a better understanding of how socio-economic deprived circumstances shape daily family life and can manifest in different modes of recognizing and handling problems in everyday life; these modes in turn – in conjunction with other factors (e.g. strategies to protect the family) – facilitate or hinder families’ identification of their candidacy for FAPS. By reconstructing these modes, and their social anchoring, we found the mediators that based on the socio-economic deprivation of these families lead to most families’ limited identification of candidacy for FAPS.

Our grounded theory confirms previous findings. Persons living in poor socio-economic circumstances have limited access to health promotion and prevention services [1–5], also when it comes to services and offers specialized in family-based addiction prevention [6, 7, 27–29]. Identified barriers to accessing and utilizing health services, such as handling health as a series of minor or major crises, normalizing health problems and symptoms, and using identity protection strategies ([5], pp. 98–101), are also prevalent when it comes to FAPS, as our results demonstrate. Also previous findings showing that engagement in preventive parenting programmes is heavily influenced by parents’ awareness of a child’s problematic behaviour or symptoms in the child [23, 24] can be confirmed for socio-economically deprived families and FAPS. Our results provide detailed insights into factors and dynamics (e.g. gender-biased or age-biased parental risk assessment) that shape and influence parents’ assumption that their child is not yet concerned with substance-use topics, therefore furthering non-identification of candidacy for FAPS. Our data demonstrates, moreover, that parents are not concerned about addiction prevention as much as about other issues in everyday life because of their lack of resources and other more pressing issues with their adolescent child. The parents’ perceptions are deeply connected with the family’s socio-demographic position. Determinants influencing journeys to and uptake of health promotion and prevention offers found in other studies [2, 3, 36], such as income/financial deprivation, education, and occupational and migration status, were manifest also in our data material. Families with modes A (existentially threatening) and B (normalizing), with overall least resources regarding the social determinants mentioned, faced the most barriers to identification of their candidacy for FAPS (see Fig. 1).

By using the concept of candidacy as a sensitizing concept [54], this study is as far as we know the first study that specifies processes and circumstances of socio-economically deprived families’ identification of their candidacy for FAPS. The study therefore extends previous notions and definitions of the stage ‘identification of candidacy’. Our data reveal that this stage is not just about how people perceive their symptoms needing medical attention ([5], p. 98), not only about people determining that they need and deserve it ([42], p. 590), and not solely about people viewing themselves as legitimate candidates for certain services ([52], p. 809): Identification of candidacy for services is also strongly influenced by the way that people recognize and handle problems in everyday life.

### Implications for policy and practice

Socio-economically deprived families cannot be considered as a homogenous group, also when it comes to a (potential) engagement with FAPS, as our results demonstrate. Therefore, there is no single strategy to reach these families. Policy and practice should build on a bundle of diverse strategies that stress especially interventions on the structural and environmental levels.

Taking the reconstructed modes and the social gradient in health for granted, even a minimal improvement in income, educational attainment, and occupational status would be a benefit for socio-economically deprived families. A “Health in All Policies” [68] approach that increases overall socio-economic resources and stabilizes the life circumstances of these families (e.g. residence status) is therefore crucial. Levelling up the social gradient would not only positively impact health and social opportunities of these families in general but could also positively influence modes of recognizing and handling problems in everyday life. This in turn would facilitate families’ identification of their candidacy for FAPS, as our analysis suggests.

The reconstructed family modes of recognizing and handling problems can guide policy and practice towards appropriate preventive interventions and ways to reach the respective subgroup. Our results indicate that from modes D (worried) to A (existentially threatening), vulnerability increases concerning candidacy for FAPS. Especially for families with mode A (existentially threatening) and B (normalizing), the existing separation of the offers in treatment-related or social welfare-oriented versus prevention-related or health promotion-related offers is not functional. These families could be better reached within their already existing networks and systems of assistance (social welfare, migration authorities, etc.). Therefore, boundaries between treatment-related and prevention/health promotion-related offers should be removed. Intersectoral collaborations should be strengthened (e. g. triage capabilities to FAPS), and FAPS should be offered by social welfare, migration authorities, and so on. Offers addressing the current main problem burden of a family – for example, financial scarcity – should incorporate family-based (addiction) prevention measures. For group C (pragmatic processing), families that pragmatically recognize problems at a low threshold and deal with them mostly within the family and their close private social network, other ways of approaching them are appropriate. Gatekeepers situated close to these families (e.g. grandparents, close friends) should be sensitized and given the necessary skills to transmit health-related knowledge and support to these families. Furthermore, approaching parents as experts on their own situation and including them in offers by using participatory intervention approaches (such as a parent-led parents’ council) might be a good way to reach type C (pragmatic processing) families.

Drawing on our findings that content-wise identification of candidacy for FAPS is heavily restricted due to most families setting other thematic priorities, and that assessments of topical relevance are biased, we suggest the following strategies. In advertising FAPS, parents’ biases and interests should be anticipated by focusing on aspects of puberty and general developmental themes in adolescence, for example. Themes of substance use/misuse, especially for parents with younger children, should not be put in the foreground. When substance use/misuse or other problem issues in adolescence are named in flyers, these themes should be appropriately framed, e.g. normalized as potential problem behaviours with which every family has to deal. This is in order to avoid defensive protection strategies on the part of the families, which could lead to non-uptake of offers. Moreover, offers of FAPS should be provided through different channels (interactive vs. non-interactive; anonymous vs. non-anonymous), providers (leisure facilities, school, social work, outreach work, etc.), and persons (teachers, social workers, psychologists, peer-to-peer counsellors) due to parents’ and children’s diverse strategies for searching for information or support and their potential rejection of certain persons or places regardless of the topic of the event. Financial incentives could increase identification of candidacy with, and uptake of, services as well as tear down or at least lessen classic barriers to services (e.g. lack of childcare).

### Limitations of the study and further research directions

Tests for data saturation provided evidence that saturation was good overall. During axial and selective coding, already identified concepts reappeared and were supported by new interview data. Future research could deepen and further develop our grounded theory. We found no LGBTIQ+ families as study participants, even though we contacted a Swiss rainbow families’ association. The results should be put also in an international context, by studies on socio-economically deprived and more affluent families in other countries. Similarities and differences could be found from which we could learn when applying measures of diversity-sensitive addiction prevention on the national and international level. Whereas this study focused on recipients of services and offers, future studies with an interactionist perspective could also include professionals working in services so as to better understand processes of candidacy from both sides.

Due to our methodological approach, we cannot make any assumptions about quantities. Therefore, it would be worthwhile to test and quantify the identified modes of recognizing and handling problems in everyday life in the overall Swiss population of families living in poor socio-economic circumstances. How many families can be located with modes A (existentially threatening), B (normalizing), C (pragmatic processing), or D (worried) in what regions in Switzerland? Answers to these questions would provide guidance for policy and practices regarding where and how to invest always scarce financial and human resources in (addiction) prevention practice.

It would be of great benefit to further discuss and enrich the present results and implications in a participatory manner with the target group and with experts from the field. The rising paradigm of participatory health research could be a promising methodology here [69].

## Conclusions

We conclude that overall, identification of candidacy for FAPS, and therefore also chances of access to and utilization of services, are limited in socio-economically deprived families due to families’ modes of recognizing and handling problems in everyday life (existentially threatening, normalizing, pragmatic processing, and worried) and other influencing factors that base on the socio-economic deprivation of these families (see Fig. 1). The diversity identified in this socio-economically deprived group(s), which is sometimes conceptualized as homogenous, has implications for policy and practice: No single preventive intervention strategy can be applied. It is important to stress especially measures on the structural and environmental levels, always taking into account the social context of deprived families.

In our opinion, the results of this study have the potential to challenge existing frameworks and systems of health promotion and FAPS with socio-economically deprived groups in Switzerland and potentially also abroad. The results also contribute to a better understanding of the stage of ‘identification of candidacy’ in the framework of the concept of candidacy itself. Finally, the qualitative approach pursued in this study turned out to be successful in gaining scientifically sound knowledge that can guide policy and practice. Therefore, in investigating equity in access to health and social services, more qualitative and also participatory research should be conducted.

## Supplementary Information

**Additional file 1.** Completed COREQ checklist for the manuscript.

**Additional file 2.** Parent interview guide

**Additional file 3.** Child interview guide.

## Data Availability

The data generated for and analysed in this study are not publicly available due to the privacy and the protection of the participants. Even though the interviews have been fully anonymized (names, places, etc.), the participants’ narratives could lead to individual identification by close family, partners, friends, etc.

## Abbreviations

FAPS: Family-based addiction prevention services
SES: Socio-economic status
