# Supplementary material for: Limited access to family-based addiction prevention services for socio-economically deprived families in Switzerland: a grounded theory study
Source: Int J Equity Health. 2020 Oct 28;19:194. doi: 10.1186/s12939-020-01305-1 (PMC7594279; doi:10.1186/s12939-020-01305-1)
Supplement: Supplementary file 2 — Additional file 2. Parent interview guide [file 12939_2020_1305_MOESM2_ESM.docx]

**Einstieg ins Interview^[[1]](#footnote-1)^:**

(Wenn möglich nach einem ruhigen Ort fragen)

Vielen Dank, dass ich mit Ihnen sprechen darf.

Ich bin….

Wir interessieren uns dafür, wie Familien mit Kindern im Altern von 10 bis 14 Jahren leben; gerade wenn die Kinder älter und jugendlich werden und manchmal neue Sachen ausprobieren.

Für uns sind *alle ihre Erfahrungen und Einschätzungen wertvoll*. Sie können also frei erzählen, was Ihnen einfällt. Es geht uns darum Ihre Sicht der Dinge zu verstehen und deshalb stelle ich vielleicht manchmal auch ein bisschen komische Fragen.

Das Gespräch *dauert* etwa bis… (1.5h), wir können uns Zeit lassen.

Alles was Sie in diesem Interview erzählen wird *vertraulich* behandelt. Ihre Erzählungen und Auskünfte werden anonymisiert. Aus den Ergebnissen wird nicht ersichtlich sein, dass Sie an dieser Forschung teilgenommen haben. Falls sie genug haben und *abbrechen* möchten können Sie das jederzeit tun. (Hinweis: Einverständniserklärung)

Mit diesem *Gerät* (zeigen!) nehme ich das Gespräch auf. Ist es ok, wenn ich es jetzt einschalte? Schön, dann mache ich das jetzt.

Vielleicht mache ich zwischendurch eine paar Notizen, die sind für mich, damit ich nichts Wichtiges vergesse.

Ist das in Ordnung? Haben Sie noch Fragen? Nein; dann legen wir doch einfach los.

**Einstiegsfrage**

**Mich interessiert wie sie so leben. Was machen sie als Familie den ganzen Tag? Was beschäftigt Sie? Erzählen Sie doch einfach mal.**

Sondierungen:

- *Tagesablauf* (Wie sieht so ein typischer Tagesablauf aus in Ihrer Familie, mit allen Freuden und Sorgen? Kontexte Schule, Familie, Quartier/Freizeit usw.)
- *Ressourcen im Alltag* (Was läuft gut? Auf was für Stützen können Sie zurückgreifen?)
- *Belastungen und Probleme im Alltag* (Wo ist es manchmal auch schwierig? Weshalb?)
- *Bewältigung von Alltagsproblemen* (Wie gehen Sie damit um? Wer/was hilft Ihnen?)
- *Umgang mit knappen sozioökonomischen Ressourcen (*Sie kommen ja mit weniger Geld aus als andere. Wie ist das im Alltag? Wo wird es manchmal auch schwierig? Welche Hilfemöglichkeiten gibt es?)
- *Allenfalls Migrationsthematik genauer abfragen.*

**Bewältigung von Entwicklungsthematiken**

Ihr Kind ist jetzt … Jahre alt. Was beschäftigt Sie derzeit in Bezug auf die Entwicklung von [Name Kind] (ihrem Kind)?

Sondierungen:

- *(Beginnende) Pubertätsthemen* (Wie gehen Sie damit um, dass ihr Kind langsam älter wird? Wie gehen Sie damit um, dass ihr Kind langsam erwachsen wird? Gibt es Themen, die Sie bereits jetzt beschäftigen? Welche? Wie? Umgang?)
- *Umgang Entwicklungsthemen*
  - Sie haben jetzt XY-Verhalten angesprochen, wie gehen Sie damit um?
  - Wer kümmert sich bei Ihnen um solche Angelegenheiten? Wie?
  - Inwieweit gibt es Personen oder Orte, die Sie unterstützten; wo Sie sich Hilfe holen können? Wie war das?
  - Welche Erfahrungen haben Sie mit diesem Angebot/dieser Technik gemacht?
- *Vermittlung Lebenskompetenzen^[[2]](#footnote-2)^*

Was geben Sie ihrem Kind mit, um das Leben gut zu meistern? Wie reagieren Sie, wenn ihr Kind Sorgen hat? Wie helfen Sie ihrem Kind, wenn es gestresst ist?

- *Prävention*

Wenn Sie an die Zukunft denken, was wünschen Sie sich für ihr Kind? Gibt es Dinge die Sie jetzt tun, damit ihr Kind in Zukunft…? Was?

**Gesundheit und suchtrelevantes Verhalten/Probierkonsum**

Wir haben bereits allgemein über die Entwicklung des Kindes gesprochen. Gesundheit kann ja auch ein Thema sein. Welche Fragen beschäftigen Sie hier?

Sondierungen:

- *Relevanzsetzungen* (Gibt es Dinge oder Bereiche auf die Sie besonders achten? Welche? Wie? Weshalb? => Ergründen, weshalb welche Gesundheitsbereiche aus welchen Gründen wichtig sind)
- *Ressourcen und Belastungen* (Was unterstützt die gesundheitliche Entwicklung besonders? Was schränkt bzw. belastet die Entwicklung besonders?)
- *Prävention* (Was tun Sie, damit Ihr Kind in Zukunft gesund bleibt?)
- *Probierkonsum* (Gerade, wenn Kinder langsam ins Jugendalter kommen, kann es sein, dass sie Tabak und Alkohol oder andere Sachen probieren. Wie sieht das in Ihrer Familie, bei Ihrem Kind aus? Erfahrungen? Wie stehen Sie dazu? Bestimmte Haltungen/Strategien? Umgangsweisen? Drogen vs. Alkohol, Tabak?)
- *Umgang mit gesundheitlichem und suchtrelevantem Verhalten*
  - Sie haben jetzt XY-Verhalten angesprochen, wie gehen Sie damit um?
  - Wer kümmert sich bei Ihnen um solche Themen? Wie?
  - Inwieweit gibt es Personen oder Orte, die Sie unterstützten; wo Sie sich Hilfe holen können? Wie war das?

**Inanspruchnahme von Gesundheitsförderung und Suchtprävention**

Manchmal gibt es ja auch Angebote die Eltern und Familien bei der Erziehung und Begleitung der Kinder unterstützen. Kennen Sie da Personen oder Angebote? (Kurse, Elternabende, Kontakte zu Fachpersonen wie Lehrer/innen, Schulsozialarbeitende, Quartierverantwortliche, Onlineforen)

Sondierungen:

- Prozesse „*Identification of Candidacy*“ (Wo waren Sie schon dabei? Wo nicht? Aus welchen Gründen? Wie waren die Erfahrungen? Was sind Themen/Angebote, die Sie interessieren (würden)?)
- *Angebote zur Stärkung von Erziehungskompetenzen* (Es gibt verschiedene Angebote, die Eltern stärken, die Kinder gut zu begleiten. Wie sind Ihre Erfahrungen dazu? – falls die Befragten dazu nichts sagen können: Beispielsweise geht es in solchen Angeboten darum, wie Eltern die Kinder gut erziehen können (Regelsetzung, Umgang mit Konflikten usw.). Wie sind Ihre Erfahrungen dazu?)
- *Angebote (Sucht-)Prävention* (Es gibt ja Personen und Organisationen, die Eltern und Familien darin unterstützten, dass Kinder gesund bleiben, keine Schwierigkeiten im Jugendalter haben, z. B. keine Probleme mit Medienkonsum, Alkohol, Tabak und Cannabis entwickeln; Kennen Sie da Angebote? Wo waren Sie schon dabei? Wo nicht? Aus welchen Gründen? Wie waren die Erfahrungen? Was sind Themen/Angebote, die Sie interessieren (würden)?
- *Verständnis Suchtprävention* (Manche Angebote laufen unter dem Titel „Suchtprävention“. Was bedeutet für Sie Suchtprävention? Ist es etwas, das für Sie oder Ihre Familie interessant ist/sein könnte? Weshalb ja/nein?)
- (Bei Bedarf, falls Gespräch nicht zum Laufen kommt: *Beispiel Angebot für Eltern* «[Was soll das Theater?](Flyer_Was_soll_das_Theater-web.pdf)» zeigen: Was denken Sie, wenn Sie das lesen? Warum? Was hat Sie angesprochen? Was nicht? Was würde sie mehr ansprechen?)

**Schluss des Interviews**

Nun haben wir viel besprochen. Das war wirklich sehr spannend. Wie Sie gegen Schluss des Interviews gemerkt haben, geht es uns auch darum, inwieweit Familien Angebote der Prävention und Gesundheitsförderung wahrnehmen oder nicht. Möchten Sie dazu noch etwas sagen?

Gibt es ganz allgemein noch etwas, das wir noch nicht besprochen haben, das für Sie aber wichtig ist? Bitte teilen Sie es mir mit. Ich möchte möglichst vollständig Ihre (Lebens-)Situation erfassen.

Vielen Dank das war wirklich spannend. Wir versuchen ja immer auch neue Familien zu finden, die mitmachen und mich würde noch interessieren, was sie motiviert hat bei uns mitzumachen?

**Kurzfragebogen – Geschlossene Fragen**

Nun möchte ich noch ein paar letzte Fragen stellen und die Angaben zur Ihrer Person aufnehmen. Wie zu Beginn erwähnt, werden auch diese Angaben vertraulich behandelt. Sie können die Fragen kurz und bündig beantworten

***Monitoring*^[[3]](#footnote-3)^**

*Wie würden Sie folgenden Aussagen einschätzen:*

Wir setzten/ Ich setze unserem/meinem Kind klare Regeln darüber was sie zuhause tun dürfen und was nicht.

Fast immer Meistens Manchmal Selten Nie

Wir setzten/ Ich setze unserem/meinem Kind klare Regeln darüber was sie ausserhaus tun dürfen und was nicht.

Fast immer Meistens Manchmal Selten Nie

Ich weiss/ Wir wissen mit wem unser/mein Kind nach der Schule/Abends unterwegs ist.

Fast immer Meistens Manchmal Selten Nie

Ich weiss/Wir wissen wo sich unser/mein Kind nach der Schule/Abends aufhält.

Fast immer Meistens Manchmal Selten Nie

***„Checkliste“***

***Substanzkonsum Familie/Kind***

- Konsumieren Sie oder Ihr/e Partner/in Alkohol, Tabak, Cannabis oder anderes?
- Wenn ja, was?
- Haben Ihr/e Kinder schon einmal Alkohol, Zigaretten oder so ausprobiert?
- In welchem Alter? Was?
- Wie häufig kommt dies etwa vor? (Regelmässigkeit eruieren)

***Inanspruchnahme Suchtprävention***

- Haben Sie in den letzten drei Jahren Angebote zum Medienkonsum von Jugendlichen /Kindern besucht? Welche?
- Haben Sie in den letzten drei Jahren Angebote zu Alkohol, Tabak, Cannabis etc. besucht? Welche?
- Haben Sie in den letzten drei Jahren Angebote zur Erziehung von Kindern und Jugendlichen besucht? Welche?
- Welche Angebote waren dies? (Titel; Inhalt)
- Wann haben Sie oder ihr Kind das letzte Mal an einem solchen Angebot teilgenommen? (Monats- und Jahresangabe) Welches Angebot war dies?

**Soziodemografische Daten**

|  | Partner/in 1 | Partner/in 2 |
| --- | --- | --- |
| (Name) |  |  |
| Geschlecht |  |  |
| Alter |  |  |
| Zivilstand |  |  |
| Wohnort (Kanton) |  |  |
| Höchster Bildungsabschluss^[[4]](#footnote-4)^ (Welches ist der höchste erworbene Ausbildungsabschluss?) |  |  |
| Beruf/Tätigkeitsbereich (genaue Berufsbezeichnung, Tätigkeitsbezeichnung) |  |  |
| Aktueller beruflicher Beschäftigungsgrad (0-100%) |  |  |
| Nationalität |  |  |
| (bei Nicht-Schweizer/in): Seit wann in der Schweiz? |  |  |
| Geburtstort der Eltern |  |  |
| Anzahl Kinder (falls Kinder mit versch. Partnern, Spalten trennen) |  | |
| Alter und Geschlecht der Kinder (falls Kinder mit versch. Partnern, Spalten trennen) |  | |
| (bei Nicht-Schweizer/in): Geburtsort der Kinder |  | |

Monatliches Netto-Haushaltseinkommen (in CHF) – dort wo die Kinder hauptsächlich leben (minus Steuerausgaben, Beiträge KVG obligatorische Krankenkasse, Sozialversicherungen (1./2. Säule):

- 6001.- und mehr
- 5001-6000.-
- 4001-5000.-
- 3001-4000.-
- 2001-3000.-
- 1001-2000.-
- Weniger als 1000.-

Erhalten Sie derzeit finanzielle Unterstützung vom Staat oder von Versicherungen? (Arbeitslosengeld, Sozialhilfegeld, EL Ergänzungsleistungen?, Entlastung von den Krankenkassenprämien) Wenn ja, welche?

**Abschluss Interview**

Wir führen die Gespräche mit mehreren Personen. Fällt Ihnen noch eine Familie aus Ihrem Bekanntenkreis ein, die gerne auch mit uns über das Thema sprechen würde?

Vielen Dank für die Auskünfte. (Übergabe Geschenk) Möchten Sie über die Ergebnisse der Studie informiert werden? (Aufnahme E-Mail oder Postadresse)

**Anhang**

Liste niederschwelliger Angebote von Beratungs- und Präventionsstellen

1. (Erhebungsinstrument: Problemzentriertes Interview) [↑](#footnote-ref-1)
2. Selbstwahrnehmung, Gefühlsbewältigung, Empathie, Kritisches Denken, Kreatives Denken, Stressbewältigung, Kommunikationsfertigkeit, Entscheidungsfertigkeit, Problemlösefertigkeit, Beziehungsfähigkeit [↑](#footnote-ref-2)
3. Auf der Grundlage von ESPAD Group (2016): ESPAD Report 2015. Results from the European School Survey Project on Alcohol and Ohter Drugs. European Monitoring Centre for Drugs and Drug Addiction (EMCDDA) and the European School Survey Project on Alcohol and Other Drugs (ESPAD). Luxemburg. [↑](#footnote-ref-3)
4. Aufnahme der genauen Ausbildungsbezeichnung und Einordnen in

   - obligatorische Schule

   - Sekundarstufe II

   - Tertiärstufe

   (Einordnung anhand der Systematik des BfS 2016: «Bildungsabschlüsse») [↑](#footnote-ref-4)
