# Supplementary material for: Limited access to family-based addiction prevention services for socio-economically deprived families in Switzerland: a grounded theory study
Source: Int J Equity Health. 2020 Oct 28;19:194. doi: 10.1186/s12939-020-01305-1 (PMC7594279; doi:10.1186/s12939-020-01305-1)
Supplement: Supplementary file 3 — Additional file 3. Child interview guide. [file 12939_2020_1305_MOESM3_ESM.docx]

**Einstieg ins Interview**

Vielen Dank, dass ich mit dir sprechen darf.

Ich bin….

(Wer bist du? (falls bislang Kommunikation nur über die Eltern gelaufen ist))

Wir interessieren uns dafür, wie du mit deiner Familie lebst; wie du aufwächst und was dich so beschäftigt.

Für uns sind alle deine Erfahrungen und Einschätzungen wichtig. Es geht uns darum deine Sicht der Dinge zu verstehen. Du kannst also frei erzählen, was dir einfällt.

Das Gespräch dauert etwa bis… (45min), wir können uns Zeit lassen.

Alles was du in diesem Interview erzählst wird vertraulich behandelt. Deine Antworten werden anonymisiert. Aus den Ergebnissen wird nicht ersichtlich sein, dass du an dieser Forschung teilgenommen hast. Falls du genug hast, kannst du das Interview jederzeit abbrechen. (Hinweis: Einverständniserklärung)

Mit diesem Gerät (zeigen!) nehme ich das Gespräch auf, damit ich danach noch genau weiss, was du mir alles erzählt hast. Ist es ok, wenn ich es jetzt einschalte? Schön, dann mache ich das jetzt.

Vielleicht mache ich zwischendurch eine paar Notizen, die sind für mich, damit ich nichts Wichtiges vergesse.

Ist das in Ordnung? Hast du noch Fragen? Nein; dann legen wir doch einfach los.

**Leitfaden Interview**

**Warm-up: Ich würde gerne zuerst ein bisschen was über dich erfahren.**

- Wie alt bist du?
- In welcher Klasse bist du?
- Gehst du hier in …. zur Schule?
- Gefällt es dir dort?
- Manchmal hast du ja auch Freizeit. Was machst du da gerne?
- Machst du das mit Freunden oder mit der Familie?

Wertschätzung zeigen, damit das Kind, der/die Jugendliche merkt, dass wir ihm/ihr gerne zuhören.

**Einstiegsfrage**

**Mich interessiert wie du lebst. Was machst du so den ganzen Tag? Was beschäftigt dich? Erzähl doch einfach mal.**

Sondierungen:

- *Tagesablauf* (Wie sieht so ein typischer Tagesablauf bei dir aus, mit allen Freuden und Sorgen? Kontexte Schule, Familie, Quartier/Freizeit usw.)
- *Ressourcen im Alltag* (Was läuft gut? Auf wen oder was kannst du zählen (Unterstützung bekommen)?)
- *Belastungen und Probleme im Alltag* (Kannst du dich an eine Situation im Alltag erinnern die du blöd fandest? Weshalb? Gibt es sonst noch Sachen die du nicht gerne magst?)
- *Bewältigung von Alltagsproblemen* (Wie gehst du damit um? Wer/was hilft dir?)
- *Umgang mit knappen sozioökonomischen Ressourcen (*Ist Geld manchmal ein Thema bei euch in der Familie? Wie gehen deine Eltern damit um? Kriegst du manchmal auch ein wenig Taschengeld? Wie viel? Was kaufst du dir davon?)

**Bewältigung von Entwicklungsaufgaben und suchtrelevantes Verhalten/Probierkonsum**

Du bist jetzt XY Jahre alt. Du bist…/Bald bist du ein/e Jugendliche/r. Hat sich etwas zu früher verändert, als du noch ein Kind warst? Was?

Sondierungen:

- *(Beginnende) Pubertätsthemen (Begriff Pubertät zurückhaltend verwenden)*
  - *Eltern-Kind-Beziehung*: Was machst du so mit deinen Eltern? Zu Hause? In der Freizeit? Nerven die manchmal auch?
  - *Peers*: Triffst du dich ab und zu mit Freunden oder Kollegen? Wo? Was macht ihr so? Gibt es in der Gruppe manchmal auch Probleme? Welche? Gibt es Kollegen, die schon Zigis, Alkohol oder anderes ausprobieren?
  - *Substanzen*: Hast du schon mal Tabak, Alkohol oder ähnliches ausprobiert? Welche? Warum? Kennst du Personen in deinem Umfeld, die dies tun? Was denken deine Eltern dazu? Sprechen sie mit dir über solche Themen? Hast du sie schon einmal gefragt? Gibt es Fragen, die du zu diesen Substanzen hast? Welche? Wer könnte dies beantworten? (Fragen aufnehmen und am Schluss des Interviews beantworten bzw. auf Ansprechpersonen verweisen)
  - *Gesundheit/Körper*: Was machst du um gesund zu bleiben? Du wirst ja älter und entwickelst dich weiter. Tauchen da auch Fragen auf? Vielleicht auch gesundheitlicher Natur? Welche? Wer könnte dies beantworten? (Fragen aufnehmen und am Schluss des Interviews beantworten bzw. auf Ansprechpersonen verweisen)
- *Umgang mit (Entwicklungs-)Problemen*
  - Du hast jetzt XY-Thema angesprochen; wie gehst du damit um? Besprichst du solche Sachen mit jemandem? Mit wem? Wenn nein, warum nicht?
  - Inwieweit gibt es Personen oder Orte (z. B. Schule, Schulsozialarbeit, Sportverein), die dir eine Hilfe sind, dir eine Antwort auf deine Frage/n geben könnten?
  - Hast du an solchen Orten schon mal angefragt? Wie war das? Würdest du wieder dort hingehen? Weshalb ja, nein?

**Inanspruchnahme von Gesundheitsförderung und Suchtprävention**

Kennst du Personen und Angebote wo du hingehen kannst, mit diesen neuen Fragen die dich beschäftigen? Z. B. Fragen zu deiner Gesundheit, zu deinem Körper oder zu Tabak, Alkohol und so?

Sondierungen:

- Prozesse „*Identification of Candidacy*“ (Wo warst du schon dabei? Wo nicht? Aus welchen Gründen? Wie waren die Erfahrungen? Was sind Themen/Angebote, die du gerne hast/haben würdest, die dich ansprechen (würden)? Was denkst du, müsste man dort beantworten oder lernen?)
- *Lebenskompetenzen:* Was machst du, wenn du mal gestresst bist? Hast du in der Schule, im Verein oder sonst wo schon einmal etwas gelernt wie man das Leben gut meistert, wie man mit Gruppendruck/Stress umgeht usw. (Lebenskompetenzen oder Selbst- und Sozialkompetenzen)? Wenn ja, wie war das? Was hat dich angesprochen? An was kannst du dich noch besonders gut erinnern? Was haben die dort vergessen zu sagen (was du gerne gewusst hättest)? Welche Kontakte, Angebote gab es sonst noch zu diesen Themen?)
- *Angebote (Sucht-)Prävention* (Habt ihr in der Schule oder anderswo schon mal über Medien- und Handykonsum, oder über Rauchen und Alkohol gesprochen? Wenn ja, wie war das? Was hat dich angesprochen? An was kannst du dich noch besonders gut erinnern? Was haben die dort vergessen zu sagen (was du gerne gewusst hättest)? Welche Kontakte, Angebote gab es sonst noch zu diesen Themen?)
- *Verständnis Suchtprävention und Prävention* (Manche dieser Angebote laufen unter dem Titel „Suchtprävention“ oder „Prävention“. Was bedeutet für dich Suchtprävention oder Prävention? Ist es etwas, das für dich interessant ist, sein könnte? Weshalb ja/nein?)

**Übergang**

Das ist wirklich spannend, danke.

Wie du gemerkt hast, geht es uns auch darum, an welchen Orten Menschen lernen können, bestmöglich gesund zu bleiben (z. B. Umgang mit Tabak, Alkohol usw.). Kommt dir dazu gerade was in den Sinn? Möchtest du dazu gerade noch etwas sagen?

Oder gibt es sonst noch etwas, das du gerne sagen möchtest?

**Kurzfragebogen – Geschlossene Fragen**

Wir sind jetzt beim letzten Teil und hier kannst du ruhig die Fragen auch kurz und bündig beantworten. Wie zu Beginn erwähnt werde ich deine Antworten nicht deinen Eltern, Freunden, Lehrern usw. weitererzählen. Das wird vertraulich behandelt.

***Monitoring^[[1]](#footnote-1)^***

Wie schätzt du folgende Situationen ein:

Meine Eltern/Vater/Mutter setzten/setzt mir klare Regeln darüber, was ich zuhause tun darf und was nicht.

☐Fast immer ☐Meistens ☐Manchmal ☐Selten ☐Nie

Meine Eltern/Vater/Mutter setzten/setzt mir klare Regeln darüber, was ich draussen tun darf und was nicht.

☐Fast immer ☐Meistens ☐Manchmal ☐Selten ☐Nie

- Was sind das für Regeln?
- Hältst du dich daran?

Meine Eltern/Vater/Mutter wissen/weiss mit wem ich nach der Schule/abends unterwegs bin.

☐Fast immer ☐Meistens ☐Manchmal ☐Selten ☐Nie

Meine Eltern/Vater/Mutter wissen/weiss, wo ich mich nach der Schule/abends aufhalte.

☐Fast immer ☐Meistens ☐Manchmal ☐Selten ☐Nie

- Setzen deine Eltern (ev. in Absprache mit dir) eine Zeit, an der du abends zu Hause sein musst? Welche Zeit? Wie funktionieren solche Abmachungen?

__________________________________________________________________________________

***Substanzkonsum Kind***

- Hast du schon einmal Tabak, Alkohol oder Ähnliches ausprobiert?
- Wenn ja, welche? Wann?
- Wie häufig kommt dies etwa vor? (Regelmässigkeit eruieren)
- Hast du in den letzten dreissig Tagen Tabak, Alkohol oder Ähnliches konsumiert? Wenn ja, welche? Wie oft? Wie viel?

***Inanspruchnahme Suchtprävention***

- Habt ihr schon einmal in der Schule oder anderswo (Verein, zuhause, Quartier, Hort) darüber geredet, wie man sein Leben gut meistert (*Lebenskompetenzstärkung*)?
- Wenn ja: Wo? Was war das Thema? Waren es mehrere Stunden/Lektionen?
- Habt ihr in der Schule oder anderswo schon mal über Tabak, Alkohol usw. geredet?
- Wenn ja: Was war das für eine Situation? Was war das Thema? Waren es mehrere Stunden/Lektionen?
- Habt ihr in der Schule oder anderswo schon mal über Medien und Internet geredet?
- Wenn ja: Was war das für eine Situation? Was war das Thema? Waren es mehrere Stunden/Lektionen?
- Wann hast du das letzte Mal an einem solchen Angebot teilgenommen? (Monats- und Jahresangabe)
- Welches Angebot war dies?

**Soziodemografische Daten**

| (Name) |  |
| --- | --- |
| Geschlecht |  |
| Alter |  |
| Wohnort (Kanton) |  |
| Schulklasse |  |
| Schultyp |  |
| Nationalität: |  |

**Abschluss Interview**

Das war sehr spannend mit dir. Als letztes habe ich noch eine Frage, was denkst du, was hat deine Eltern dazu motiviert bei diesem Gespräch mitzumachen?

- Abgabe Infoblatt Angebote (mit den Kindern und Jugendlichen anschauen und auf für sie relevante Angebote hinweisen)
- Vielen Dank für die Auskünfte. (Übergabe Geschenk)
- Fällt dir noch ein Freund/ Kollege ein, mit dem wir sprechen könnten?

**Anhang**

Liste niederschwelliger Angebote von Beratungs- und Präventionsstellen

1. Auf der Grundlage von ESPAD Group (2016): ESPAD Report 2015. Results from the European School Survey Project on Alcohol and Ohter Drugs. European Monitoring Centre for Drugs and Drug Addiction (EMCDDA) and the European School Survey Project on Alcohol and Other Drugs (ESPAD). Luxenburg. [↑](#footnote-ref-1)
